# Severe Hypernatremia in Combined Diabetic Ketoacidosis and Hyperglycemic Hyperosmolar State: A Case Report of Two Japanese Children

**DOI:** 10.7759/cureus.9672

**Published:** 2020-08-11

**Authors:** Saho Shima, Satoko Umino, Miyuki Kitamura, Kikumi Ushijima, Shuichi Yatsuga

**Affiliations:** 1 Pediatrics and Child Health, Kurume University School of Medicine, Kurume, JPN

**Keywords:** type 1 diabetes mellitus, diabetes ketoacidosis, hyperglycemic hyperosmolar state, hypernatremia

## Abstract

As sodium level in diabetic ketoacidosis (DKA) and hyperglycemic hyperosmolar state (HHS) is usually low, normal, or slightly elevated, severe hypernatremia with DKA and/or HHS is rare. Case 1 was a 14-year-old boy, presenting with typical laboratory test values and symptoms consistent with DKA and HHS. His corrected sodium level, 172 mEq/L, might have occurred as a result of consuming 6 L/day of highly carbonated, carbohydrate- and sodium-rich drinks during the week preceding the diagnosis. This patient developed right lung artery thrombosis, which did not require treatment. Case 2 was a 10-year-old girl, presenting with typical laboratory test values and symptoms of DKA and HHS. Her corrected sodium level, 175 mEq/L, might have occurred as a result of large electrolyte-free water loss associated with osmotic diuresis. These two cases of patients presenting with DKA-HHS and severe hypernatremia are the first to be reported in Japan.

## Introduction

Diabetic ketoacidosis (DKA) and hyperglycemic hyperosmolar state (HHS) are complications that present in the acute phase of diabetes mellitus (DM). They are associated with increased risk of mortality [1-2]. DKA presents in patients with type 1 DM (T1DM), while HHS is more commonly seen in patients with type 2 DM (T2DM) [3]. DKA is defined with the following laboratory values: 1) blood glucose (BG) ≥ 200 mg/dL, 2) pH < 7.30, 3) HCO3- < 15 mmol/L, and 4) ketonuria and/or ketonemia [4].

Hyperglycemic hyperosmolar state is defined based on the following laboratory values: 1) BG > 600 mg/dL, 2) venous pH > 7.25 and/or arterial pH > 7.30, 3) HCO3- > 15 mmol/L, 4) mild ketonuria or absent to mild ketonemia, 5) plasma osmolarity > 320 mOsm/kg, and 6) altered level of consciousness (obtundation, combativeness) or seizures [4]. HHS is sometimes accompanied by mild to moderate acidosis due to severe dehydration, including lactic acidosis due to hypoperfusion [4]. Symptoms of DKA and HHS can overlap. Combined DKA-HHS syndrome is defined by the following laboratory values: 1) plasma osmolarity ≥ 320 mOsm/kg, 2) BG ≥ 600 mg/dL, 3) pH ≤ 7.30, and 4) ketonuria and/or ketonemia [5].

To date, few cases of pediatric DKA-HHS with severe hypernatremia have been only reported, as DKA tends to present with hyponatremia or eunatremia, while HHS tends to present with hyponatremia, eunatremia, or mild hypernatremia due to sodium chloride excretion [1]. In 2014, the first case of DKA-HHS with severe hypernatremia in the Asian population was reported [2]. Herein, we report the first two Japanese pediatric cases of hypernatremia in DKA-HHS.

## Case presentation

Case 1

Case 1 was a 14-year-old Japanese boy who had been prescribed methylphenidate hydrochloride for attention deficit hyperactivity disorder (ADHD). He was born at 40 weeks of gestational age, with a birth weight of 2690 g (-1.2 SD) and birth length of 46.4 cm (-1.7 SD). He had a height of 172.0 cm (+1.35 SD), body weight of 63.0 kg (+1.0 SD), and body mass index (BMI) of 21.3 kg/m2 (+0.6 SD). His body weight was 72.0 kg a few weeks before admission. There was no family history of DM. One month before the diagnosis of DM, the patient had polydipsia and thirst. In the week preceding the diagnosis, he consumed over 6 L/day of a sports drink (Na; 15 mEq/L) and 2.5 L/day of milk (Na; 22 mEq/L). He presented with generalized fatigue and received 0.5 L/day intravenous drip (DIV) of 0.9% saline with 2.6% dextrose (Na; 154 mEq/L) continuously for two days for thirst, vomiting, and fatigue. However, his symptoms did not improve and therefore, was referred to our hospital. He was restless but able to maintain conversation. His vital signs are shown in Table 1. Clinical examination revealed a fruity breath odor, Kussmaul breathing, and lethargy. A diagnosis of DKA-HHS was made based on the levels shown in Table 1.

**Table 1:** Vital signs and laboratory data of the two cases. ND, not detectable; BE, base excess; RBC, red blood cell; Hb, hemoglobin; Hct, hematocrit; WBC, white blood cell; Plt, platelet; AST, aspartate aminotransferase; ALT, alanine aminotransferase; LDH, lactate dehydrogenase; BUN, blood urea nitrogen; Cr, creatinine; UA, uric acid; Amy, amylase; T-chol, total cholesterol; CK, creatine kinase; Glu, glucose; HbA1c, hemoglobin A1c; Ab, antibody; GAD, glutamic acid decarboxylase; IA-2, insulinoma-associated antigen-2; IRI, immunoreactive insulin; CRP, C-reactive peptide; CPR, C-peptide immunoreactivity; u, urine

Indicator		Case 1	Reference range	Case 2	Reference range
Systolic blood pressure	mmHg	123		111	
Diastolic blood pressure	mmHg	60		62	
Heart rate	beats per minute	143		128	
Respiratory rate	breaths per minute	143		32	
Body temperature	ºC	37.4		36.9	
pH		7.12	7.35-7.45	7.073	7.35-7.45
PCO_2_	mmHg	22.4	35-48	34.3	32-45
HCO_3_^-^	mmol/L	7.1	22-29	9.8	22-29
BE	mmol/L	-20.1		-19.4	
RBC[A1]	x10^6^/µL	6.21	4.5-5.3	5.34	4.0-5.2
Hb	g/dL	18.3	13.0-16.0	15.7	11.5-15.5
Hct	%	52.6	35-45	51.1	35-45
WBC	x10^3^/µL	14.6	4.5-13.5	21.2	4.5-13.5
Plt	x10^3^/µL	258	150-400	402	150-400
AST	U/L	20	5-45	10	5-45
ALT	U/L	33	5-45	21	5-45
LDH	U/L	213	230-460	206	246-497
BUN	mg/dL	44	7-18	55	7-18
Cr	mg/dL	1.59	0.5-1.0	1.71	0.5-1.0
UA	mg/dL	15.5	3.0-7.7	13.4	2.2-6.6
Na	mmol/L	146	136-146	159	136-146
K	mmol/L	5.4	3.5-5.0	4.5	3.5-5.0
Cl	mmol/L	97	98-106	115	98-106
Ca	mg/dL	8.8	8.4-10.2	10.1	8.4-10.2
P	mg/dL	3.2	2.9-5.4	5.8	3.7-5.6
p-Osm	mOsm/kg	363	275-295	418	275-295
Amy	U/L	45	30-100	74	30-100
T-Chol	mg/dL	407	130-204	216	124-217
TG	mg/dL	2797	36-138	170	35-114
CK	U/L	1240	5-130	38	5-130
Glu	mg/dL	1045	60-100	1070	60-100
HbA1c	%	12.3	3.0-6.2	15.2	3.0-6.2
Serum total ketone	µmol/L	> 10000	<100-500	13686	<100-500
Anti-insulin Ab	U/mL	< 0.4	<0.4	< 0.4	<0.4
Anti-GAD Ab	U/mL	< 5.0	< 5.0	< 5.0	< 5.0
IA-2 Ab	U/mL	< 0.6	< 0.6	1.7	< 0.6
IRI	µU/mL	3.7	7-24	21.1	7-24
CPR	ng/mL	2.0	0.4-2.2	0.4	0.4-2.2
Urine CPR (3 days)	µg/day	0.7/2.9/24.7	22.8-155.2	4.6/16.7/49.1	22.8-155.2
u-specific gravity		1.036	1.002-1.030	ND	1.002-1.030
u-glucose	mg/dL	≥1000		ND	
u-ketone		4+	-	ND	-
u-blood		3+	-	ND	-
u-protein		±	-	ND	-
u-pH		≤5.0	4.5-8	ND[A2]	4.5-8

**Figure 1:**
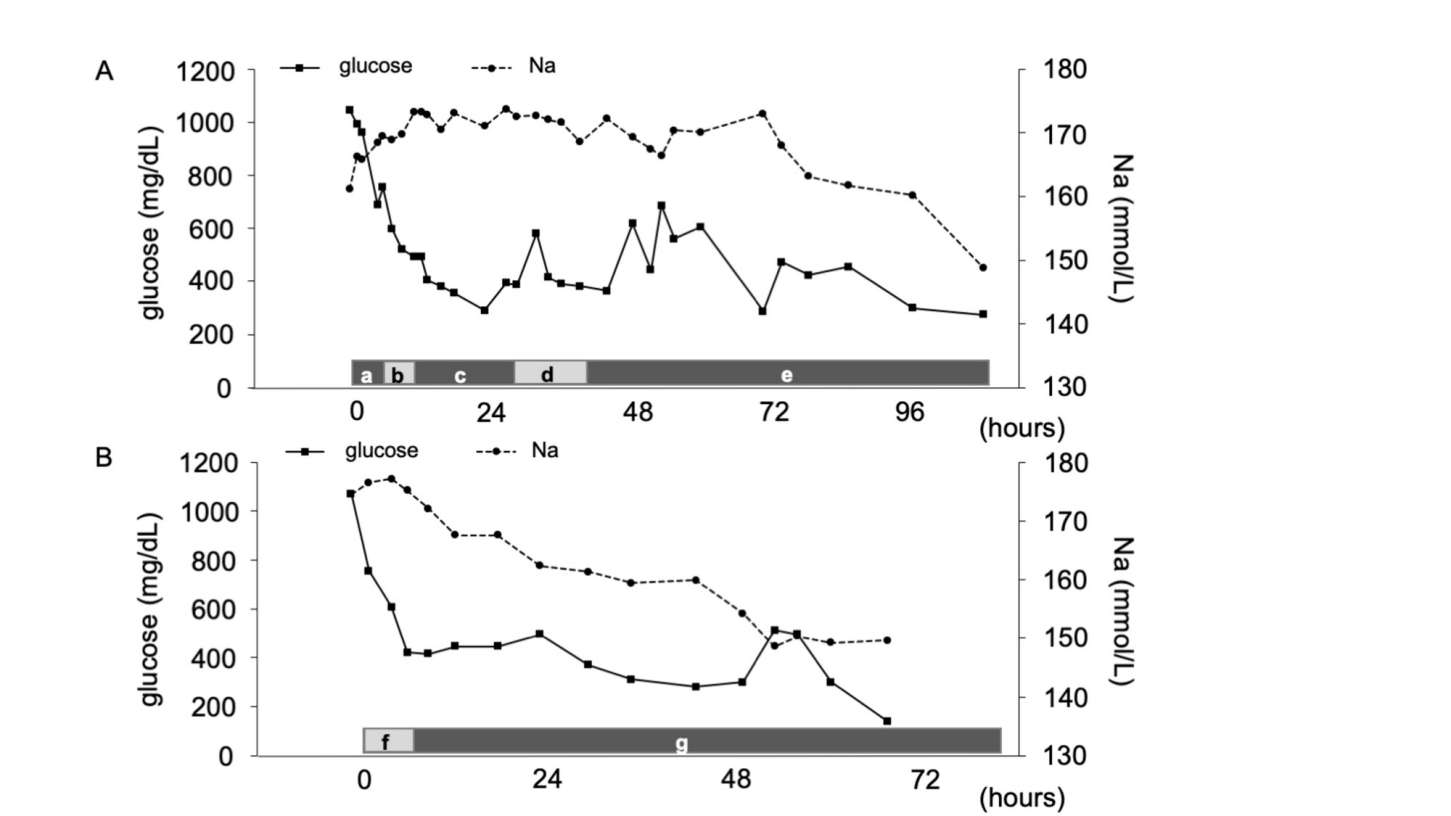
Trend of glucose and sodium level during correction. A. Case 1, a: 0.9% saline for 2.5 L in 5 hours; b: 0.58% saline with potassium and glucose for 1.0 L in 5 hours; c: 0.19% saline with potassium and glucose for 1.4 L in 17 hours; d: glucose 5% water including potassium for 0.9 L in 11 hours; e: 5% dextrose water with potassium or 0.19%-0.58% saline with potassium in accordance with sodium levels. B. Case 2, f: 0.9% saline for 1.4 L in 7 hours; g: 0.18%-0.53% saline including potassium and glucose.

We administered 0.58%-0.9% saline and insulin at 0.05 U/kg/h via DIV. Finally, his sodium level returned to the normal range after 112 hours from admission (Figure 1A). On admission, creatine kinase (CK) was elevated at 1240 U/L. While the urine occult blood was 3+, the urine was negative for microscopic red blood cells, suggesting asymptomatic rhabdomyolysis as myalgia was absent. On day three, he complained of chest pain. His platelet count was 8.3 × 104/µL (reference range, rr: 13104-36 × 104/µL) and fibrin/fibrinogen degradation product (FDP) was 16.5 µg/mL (rr: <5 µg/mL). An enhanced whole-body CT scan detected thrombosis of the right pulmonary artery, pneumomediastinum, and subcutaneous emphysema. These symptoms spontaneously remitted. The patient did not develop any neurological complications. 

One year postadmission, the patient has a height of 172.8 cm (+0.9 SD), body weight of 80.7 kg (+2.2 SD), and BMI of 27 kg/m2 (+1.7 SD). His treatment consists of 24 units/day of insulin degludec and 35 units/day of insulin aspart (0.73 units/body weight/day). He was diagnosed with T1DM, complicated with DKA-HHS and severe hypernatremia due to soft drink ketosis.

Case 2

Case 2 was a 10-year-old Japanese girl, with no significant past medical history. She was born at 39 weeks of gestational age, with a birth weight of 3202 g (+1.1 SD) and birth length of 49.5 cm (+0.4 SD). She had a height of 148.6 cm (+1.8 SD), body weight of 26.2 kg (-0.9 SD), and BMI of 11.9 kg/m2 (-3.6 SD). Her body weight was 31.1 kg one month prior to presentation There was no family history of DM. She was referred to our hospital after presenting with generalized fatigue, polydipsia, and polyuria. She was able to maintain conversation but was moderately restless in general. Her vital signs are shown in Table 1. A clinical examination revealed fruity breath odor, Kussmaul breathing, and lethargy. A diagnosis of DKA-HHS was made based on her laboratory findings (Table 1).

We administered 0.58%-0.9% saline and insulin 0.05 U/kg/h via DIV. Finally, her sodium level returned to the normal range after 78 hours from admission (Figure 1B). She did not develop any neurological complications. 

Six months postadmission, the patient is 146.4 cm (+1.2 SD) tall, with a body weight of 33.4 kg (-0.6 SD) and BMI of 13.6 kg/m2 (-2.1 SD). Her treatment consists of 10 units/day of insulin degludec and 19 units/day of insulin lispro (0.87 units/body weight/day). She was diagnosed with T1DM, complicated with DKA-HHS and severe hypernatremia.

## Discussion

To the best of our knowledge, this is the first report of two pediatric cases of DKA-HHS with severe hypernatremia, including a case with complications of asymptomatic rhabdomyolysis, thrombosis of right pulmonary artery, pneumomediastinum, and subcutaneous emphysema simultaneously, in Japan.

Diabetic ketoacidosis-hyperglycemic hyperosmolar state occurs in 13.8% of pediatric hyperglycemic emergencies [5]. It is not usually accompanied by hypernatremia as hyperglycemia dilutes the serum sodium concentration by shifting water from the intracellular space to the extracellular space. Despite large water losses, sodium level is usually low or normal in patients with DKA. The level can be low, normal, or slightly elevated in HHS due to the water shift [1]. Hypernatremia in new-onset T1DM with DKA can be caused by the consumption of large amounts of carbohydrate-rich beverages to quench thirst [6]. 

Hyperglycemic hyperosmolar state tends to present with hyponatremia, eunatremia, or slight hypernatremia, as HHS is induced by dilution of extracellular fluid and/or dehydration of osmotic diuresis. DKA may present with mild hypernatremia due to severe dehydration. However, exact mechanism of severe hypernatremia is unknown [7]. The mechanism of severe hypernatremia in patients with new-onset DM is proposed that the increased proximal renal tubular sodium reabsorption due to negative feedback of hyperinsulinemia may be exceptionally efficient and in sodium retention [8]. The first Asian pediatric case was reported in Korea in 2014. The patient had consumed a large amount of sports drink, which contained high volume of sugar, sodium, and carbonate that might have exacerbated the hypernatremia [3]. The patient in Case 1 also had consumed a high volume of sports drinks and milk for one week, likely leading to the exacerbation of symptoms and severe hypernatremia at the onset of DKA-HHS. However, the patient in Case 2 had no history of such intake. The extreme hypernatremia might have occurred primarily as a result of the large electrolyte-free water losses associated with osmotic diuresis [9]. Electrolyte imbalance tends to be more severe in HHS than in DKA because osmotic diuresis tends to last longer in HHS [10]. Osmotic diuresis with hyperglycemia and ingestion of high amount of sodium might exacerbate hypernatremia in these cases.

Among the severe complications associated with DKA is brain edema. Slow correction of hyperglycemia and hypernatremia lowers the risk of brain edema [3]. In pediatric patients, the treatment approach to DKA and DKA-HHS is similar. However, DKA-HHS requires administration of a greater amount of fluid and careful monitoring of hemodynamics [5]. There are currently no guidelines regarding treatment of pediatric and/or adolescent HHS [4]. As we carefully monitored patient symptoms, glucose level, and electrolyte level, we believe that neither of the patients presented developed neurological complications.

The present cases were difficult to diagnose. Case 1 was diagnosed with T2DM because insulin-related antibodies were all negative, and 24 hours urine C-peptide did not show absence of insulin. However, he was not obese and required insulin therapy (0.73 units/body weight/day), indicating that he may have slowly progressive T1DM. Case 2 was diagnosed with T1DM because the IA-2 antibody was weakly positive. She was also not obese and required insulin therapy (0.87 units/body weight/day). However, 24 hours urine C-peptide showed presence of insulin. She may still have T2DM, but further observation is necessary.

## Conclusions

We report the first two Japanese pediatric cases of DKA-HHS with severe hypernatremia. DKA-HHS results in more complications than only DKA or HHS. The findings from these cases should be considered to address the possibility of pediatric patients developing rarer complications of diabetes, including rhabdomyolysis, thrombosis, pneumomediastinum, and subcutaneous emphysema. We believe that careful fluid therapy in DKA-HHS prevents severe life-threatening complications such as brain edema or death, although several complications except for the life-threatening ones may occur.
